# High-Sensitivity C Reactive Protein: Associations with Cardiovascular Risk Factors and Tracking in Female Adolescents and Young Adults

**DOI:** 10.5402/2011/707206

**Published:** 2011-01-24

**Authors:** John A. Morrison, Charles J. Glueck, Stephen R. Daniels, Ping Wang, Davis M. Stroop, Paul S. Horn

**Affiliations:** ^1^Division of Cardiology, Cincinnati Children's Hospital Medical Center, Cincinnati, OH 45229-3039, USA; ^2^Cholesterol Center, Jewish Hospital of Cincinnati-Mercy Health Partners, ABC Building, 3200 Burnet Avenue, Cincinnati, OH 45229, USA; ^3^Children's Hospital, Denver, CO 80045, USA; ^4^Division of Hematology, Cincinnati Children's Hospital Medical Center, Cincinnati, OH 45229-3039, USA; ^5^Department of Mathematical Sciences, University of Cincinnati, Cincinnati, OH 45221, USA; ^6^Psychiatry Service, Veterans Affairs Medical Center, Cincinnati, OH 45220, USA

## Abstract

*Objective*. We assessed adolescent anthropometry, lipids, insulin, glucose, and blood pressures to identify factors associated with high-sensitivity C-reactive protein (hsCRP) and its tracking in young adults. *Methods*. Ten-year prospective study of 589 schoolgirls, 321 black, 268 white. *Results*. HsCRP did not differ (*P* > .08) by race or oral contraceptive use. HsCRP tracked from age 16 to 25 (*r* = 0.77), 16 to 26 (*r* = 0.50), 24 to 26 (*r* = 0.66), and 25 to 26 (*r* = 0.71), all *P* ≤ .02. By stepwise regression, at age 16, waist circumference accounted for 44.8% of hsCRP variance; BMI accounted for 33.1%, 34.4%, and 31.1% at ages 24, 25, and 26, *P* < .0001 for all. Changes in cholesterol and BMI were associated with change in hsCRP from age 24–26 (partial *R* ^2^ = 12.3%  *P* < .0001, 6.6%  *P* = .0012). Changes in BMI and triglyceride (partial *R* ^2^ = 8.5%  *P* = .0001, 3.3%, *P* = .0045) were associated with change in hsCRP from age 25 to 26. *Conclusions*. HsCRP tracks from age 16 to 26, with BMI, waist circumference, and cholesterol as major determinants.

## 1. Introduction

 The anatomic and pathophysiological genesis of atherosclerosis is in childhood [1–5]. The traditional Framingham risk factors (hyperlipidemias, blood pressure, obesity, diabetes, and cigarette smoking) have been used as the starting point for evaluating individual risk to vascular disease, and each factor is strongly associated with increased risk to coronary heart disease (CHD) in adults and, in children, associated with family history of CHD [6–9]. It has been estimated that the traditional CHD risk factors may explain about 50% of CHD morbidity and mortality [9], eliciting efforts to identify additional predictive factors, such as high-sensitivity C reactive protein (hsCRP) [10].

Biomarkers predictive of inflammatory processes in acute clinical settings (e.g., during infection or after traumas) have become valuable predictors of future vascular events [11]. This primal organism response to invading pathogens, trauma, or chronic inflammation presumably represents a protective mechanism and involves a cascade of thrombotic and inflammatory factors of variable expression [11]. In the chronic state, acute phase reactants have become important predictors of future vascular events: thus, baseline serum high-sensitivity c-reactive protein (hsCRP) values have been correlated with the development of CHD in healthy men [11, 12] and women [11] in the Harvard Study population, corroborated by other groups [13, 14]. In a prospective study of 506 men who had fatal CHD or had a nonfatal myocardial infarction compared to 1025 men free of CHD, men in the top third for entry hsCRP had an odds ratio for CHD of 2.13 (95% confidence interval 1.38–3.28) after age, town, smoking, vascular risk factors, and indicators of socioeconmic status were adjusted for [15]. The cascade of TNF-*α* and IL-6 elevation leads to increments in hsCRP and fibrinogen, with resulting adhesion molecule expression, and may facilitate development of type 2 diabetes mellitus (T2DM) as well as CHD [16].

Cross-sectional studies in children have shown a strong association between hsCRP and obesity, triglycerides, HDL cholesterol, diastolic blood pressure, and fibrinogen [17–20]. Longitudinal data from the Young Finns Study [21] showed that hsCRP tracked significantly from ages 3–18 years (at entry) to ages 24–39 years. Tracking was greater in the older children and in women than men [21]. By multivariate analysis, adult carotid artery intimal medial thickness (cIMT) was associated with childhood elevated systolic blood pressure, high LDL cholesterol, and smoking, but not with childhood hsCRP [21]. Järvisalo et al. [22], however, showed that elevated hsCRP levels in children are associated with increased cIMT as well as with decreased brachial artery flow-mediated dilatation, and Zieske et al. [23] reported that serum hsCRP is independently associated with advanced atherosclerosis in youth. Thus, in aggregate, these studies suggest that measurement of hsCRP in childhood [21–23] might predict adult cardiovascular risk. 

The National Growth and Health Study (NGHS) [24], its ancillary studies, and the extension of the followup of the Cincinnati clinic cohort by investigator-initiated research projects, provide an opportunity to assess hsCRP tracking in black and white females from mid-adolescence to young adulthood and to identify factors associated with high concentrations of hsCRP and with its tracking.

## 2. Materials and Methods

### 2.1. The Study Population

The NGHS has been described previously [24]. Briefly, NGHS was a 10-year (1987–1997) multicenter cohort study to explicate origins of black-white disparities in obesity and its effects on cardiovascular (CVD) risk factors in women [24]. Race was self-declared and enrollment was restricted to 9- and 10-year old girls from racially concordant households, that is, to girls who said they were black or white and whose parents or guardians said that they were black or white, respectively. In ancillary projects, the Cincinnati clinic measured fasting insulin at Visit 7 (mean ± SD age 16 ± 1), in addition to the NGHS variables—lipid profiles, apo A1, systolic and diastolic blood pressure [25]. At age 16, hsCRP was measured in a subset of participants as a pilot project. After completion of NGHS, the Cincinnati clinic extended followup with measurement of insulin to age 25 and collected hsCRP at ages 24, 25, and 26. 

In the NGHS [25], and its extension, procedures followed were in accordance with the ethical standards of the Institutional Review Boards of the Centers, who approved the study. Signed informed consent was obtained from the girls' parents or guardians and assent from the girls.

### 2.2. Laboratory and Clinical Measurements

Methods for measurement of lipids, fasting serum insulin, glucose, height, weight, waist circumference, and systolic and diastolic blood pressure have been previously described [24, 25]. Fasting glucose was measured at ages 10 and 19–24, insulin at ages 10, 16, and 19–25.

At each visit, girls were asked whether they were taking a birth control pill, since hsCRP may be elevated by estrogen-progestin oral contraceptives [26].

The NGHS used BMI to assess overweight annually according to a standard protocol [24], as recommended by several expert panels [27–29] and waist circumference as an indicator of fat patterning. 

 Using criteria previously described [30], NGHS subjects having fasting blood glucose ≥126 mg/dl [31] at age 10 or type 1 DM at any time from age 10 through age 25 were excluded (*n* = 7) from the analysis sample for this report. Diagnosis of diabetes was based on WHO criteria, fasting glucose ≥126 mg/dl, and self-reported diabetes with treatment by a physician [31]. We did not have measurement of C-peptides as well as diabetes autoantibody levels, gold standard methods [32] to distinguish type 1 from type 2 diabetes. 

Fasting serum insulin levels (competitive protein-binding radioimmunoassay) were measured after an overnight fast (≥8 hr) using the Michigan Diabetes Research and Training Center (Ann Arbor) at age, and using the Endocrine Lab at the University of Cincinnati/Children's Medical Center at ages 16, 19, and 20–25. 

HsCRP was measured using the N high-sensitivity latex-enhanced immunonephelometric (BN 100 nephelometer, Dade Behring) assay.

### 2.3. Statistical Methods

Since hsCRP was not normally distributed, black-white differences in hsCRP and differences between birth control pill users and nonusers were assessed by Wilcoxon rank sum tests. For assessment of changes in hsCRP and for regression analyses, hsCRP was transformed in logarithm scale.

Spearman correlations were used to assess the tracking of hsCRP from age 16 to 26 years, Table 1. After adjusting for age, race, BMI, waist circumference, insulin, maximum glucose, total cholesterol, HDL cholesterol, LDL cholesterol, triglyceride, and systolic and diastolic blood pressure, Spearman partial correlations were also calculated to assess the tracking of hsCRP from ages 24 to 26, and 25 to 26, Table 1.

Spearman correlations were used to assess the associations of hsCRP with cardiovascular risk factors at ages 16, 24, 25, and 26 years including age, race, BMI, waist circumference, insulin, total cholesterol, triglyceride, HDL cholesterol, LDL cholesterol, systolic blood pressure, diastolic blood pressure, and maximum glucose, Table 2. The Hochberg-Benjamini [33] False Discovery Rate (FDR) for multiple comparisons was used, Table 2.

After log transformation of hsCRP, all of the variables from Table 2 were incorporated as candidate explanatory variables into stepwise regression models with hsCRP as the dependent variable at ages 16, 24, 25, and 26, Table 3.

Spearman correlations were used to assess relationships of change in hsCRP (in logarithm scale) from ages 24 to 26 and from 25 to 26 with changes in cardiovascular disease variables: age, BMI, total cholesterol, triglyceride, HDL cholesterol, LDL cholesterol, systolic blood pressure, and diastolic blood pressure during the same period, Table 4. The FDR approach [33] for multiple comparisons was again used, Table 4.

All of the variables from Table 4 were incorporated as candidate explanatory variables into stepwise regression models with change in hsCRP (in logarithm scale) from age 24 to 26 and from 25 to 26 as dependent variables, Table 5.

## 3. Results

HsCRP did not differ between birth control pill users versus nonusers, *P* ≥ .28 and did not differ by race, *P* ≥ .09. Hence, data are presented for the total cohort without subcategorization by birth control pill use or race.

There were no differences (*P* > .05) in hsCRP or CVD risk factors between girls with ≥2 sequential hsCRP measurements and those with only 1 measurement.

Median and interquartile range for hsCRP at age 16 were 1.5 (0.7–5.6), at age 24, 2.0 (0.7–4.0), at 25, 1.7 (0.6–4.2), and at 26, 1.7 (0.6–4.6) mg/L. 

### 3.1. Tracking of hsCRP from Age 16 to Age 26 Years

HsCRP was measured in 589 girls, 321 black, and 268 white. Subsets of the cohort had hsCRP measured at different ages, 1, 2, 9, and 10 years apart, making possible studies of hsCRP tracking and factors associated with it, Table 1. Thus, there were 37 girls having hsCRP measured at age 16 (18 black, 19 white) with subsequent measurements at age 24 (*n* = 6), 25 (*n* = 22) and 26 (*n* = 21), Table 1. In addition, 172 girls (91 black, 81 white) had hsCRP measured at age 24, with subsequent measures at age 25 (*n* = 3) and at age 26 (*n* = 147), Table 1. Additionally, hsCRP was measured at age 25 in 391 girls (217 black, 174 white) and subsequently at age 26 in 225 of these girls, Table 1. HsCRP was measured at age 26 in 392 girls (215 black, 177 white), Table 1. 

Age 16 hsCRP correlated with subsequent measures at ages 25 (*r* = 0.77, *P* < .0001) and 26 (*r* = 0.50, *P* = .02), Table 1. Age 24 hsCRP correlated with levels at age 26 (*r* = 0.66, *P* < .0001) and age 25 hsCRP correlated with levels at age 26 (*r* = 0.71, *P* < .0001), Table 1.

After adjusting for age, race, BMI, insulin, maximum glucose, total cholesterol, triglyceride, HDL cholesterol, LDL cholesterol, and systolic and diastolic blood pressure, hsCRP at age 24 remained highly correlated with levels at age 26 (partial *r* = 0.63, *P* < .0001), as did hsCRP at age 25 with levels at age 26 (partial *r* = 0.54, *P* < .0001), Table 1.

Of 43 girls with hsCRP at age 24 in the top 30% of the distribution, 24 (56%) remained in the top 30% at age 26, while 26 of 41 (63%) with hsCRP at age 24 in the bottom 30% of the distribution remained in the bottom 30% at age 26. Of 62 girls in the hsCRP top 30% at age 25, 42 (68%) remained in the top 30% at age 26, while 49 of 72 (68%) originally in the bottom 30% at age 25 remained there at age 26.

### 3.2. Correlates of hsCRP

Of the 37 girls at age 16, significant positive bivariate correlates with hsCRP included BMI, waist circumference, insulin, and maximum glucose, Table 2. 

At ages 24, 25, and 26, significant bivariate correlates with hsCRP included BMI, waist circumference, insulin, total cholesterol, triglyceride, HDL cholesterol, LDL cholesterol, systolic blood pressure, diastolic blood pressure, and maximum glucose, Table 2. Age was significantly correlated with hsCRP at age 26. 

By stepwise regression, waist circumference (age 16, partial *R* ^2^ = 44.8%) and BMI (ages 24, 25, and 26, partial *R* ^2^ = 33.1%, 34.4%, 31.1%) were the major significant (*P* < .0001) explanatory variables for hsCRP, Table 3. At ages 24 and 25, total cholesterol levels were significant explanatory variables, accounting for 2.5% and 1.9% of hsCRP variance, Table 3. At age 26, triglyceride accounted for 3.5% of hsCRP variance, Table 3. With the exception of inclusion of race as a significant explanatory variable at age 26, the results in Table 3 were identical to results if only those variables which had significant bivariate correlations in Table 2 were included as candidate explanatory variables (data not shown).

Race was unrelated to change in hsCRP from age 24 to 26, and from age 25 to 26, Table 4. Changes in BMI, waist circumference, total cholesterol, triglyceride, and LDL cholesterol were positively and significantly related to changes in hsCRP from ages 24 to 26, Table 4. Changes in BMI, waist circumference, total cholesterol, and triglyceride were positively and significantly related to changes in hsCRP from age 25 to 26, Table 4. 

After including all of the candidate explanatory variables of Table 4 into a stepwise regression model, change in total cholesterol and change in BMI were significant explanatory variables for change in hsCRP from age 24 to 26 (partial *R* ^2^ = 12.3%, 6.6%), Table 5. Changes in BMI (partial *R* ^2^ = 8.5%) and change in TG (partial *R* ^2^ = 3.3%) were significant explanatory variables for change in hsCRP from age 25 to 26, Table 5. The results in Table 5 were identical to results if only those variables which had significant bivariate correlations in Table 4 were included as explanatory variables (data not shown).

## 4. Discussion

The chronic expression of acute phase proteins, in the stable, ostensibly nonacute setting, is of unclear etiology. Such proteins could be present in excess due to (a) incomplete resolution of a previous acute episode, (b) ongoing monocyte/macrophage activation, and/or (c) enhanced baseline production determined by coordinate genetic control. Activation of the monocyte/macrophage in the acute setting may be among the first dominoes in the inevitable cascade of expressed cytokines and growth factors [34, 35]. Activated monocytes produce TNF and IL-1-like cytokines, which lead to the full acute phase reaction, including hsCRP (17). 

In adults, statins lower hsCRP concurrently with LDL cholesterol, and hsCRP levels achieved on statin therapy are predictive of CVD event rates irrespective of the lipid endpoint used [10]. To test the hypothesis whether or not subjects with normal LDL cholesterol but elevated hsCRP represent a population at increased risk that might benefit from statin treatment, the JUPITER trial [10] randomized 17,802 apparently healthy persons with elevated hsCRP to 20 mg Rosuvastatin daily or placebo. The rates of the primary endpoint (composite of nonfatal myocardial infarction, nonfatal stroke, hospitalization for unstable angina, revascularization, and confirmed death from cardiovascular causes) were 0.77 and 1.36 per 100 person-years of followup in the Rosuvastatin and placebo groups, respectively. Relative risk reduction was 44% [10]. It was postulated that hsCRP more accurately selected high-risk subjects than LDL cholesterol due to hsCRP's association with multiple CVD risk factors, thus representing an aggregate-integrated marker of the total inflammatory burden of an individual [10]. While inflammation is a crucial component of atherothrombosis [36] and patients with elevated hsCRP and low or intermediate LDL cholesterol levels [10, 37] are at increased vascular risk, it is not known whether inhibition of inflammation per se will lower vascular event rates [36]. A direct test of the inflammatory hypothesis of atherothrombosis requires an agent that inhibits inflammation without affecting other components of the atherothrombotic process, particularly LDL cholesterol [36]. The forthcoming cardiovascular inflammation reduction trial (CIRT) will randomize 7000 stable coronary artery disease patients with persistent elevations of hsCRP to placebo or very low-dose methotrexate (10 mg weekly), a proven anti-inflammatory regimen that reduces TNF-alpha, IL-6, and CRP levels and is in wide use among rheumatoid arthritis patients [36]. The CIRT trial [36], if successful, would help confirm the inflammatory hypothesis of atherothrombosis and open innovative, anti-inflammatory approaches to the treatment and prevention of cardiovascular disorders.

It is not yet known whether hsCRP is a risk factor for cardiovascular disease independent of other known risk factors [10, 37], nor is it well understood how long hsCRP would need to be normalized independent of changes in other CVD risk factors to reduce CVD events [36]. Moreover, it is not yet known whether hsCRP, a $43 test, is useful in assessing childhood antecedents of young adult cardiovascular risk above and beyond conventional childhood CVD risk factors [21], and whether high hsCRP is reversible with childhood therapeutic interventions. Although Karakas and Koenig reported that birth control pill use elevated hsCRP [38], it had no significant effect on hsCRP in our current study. Juonala et al. [21] reported significant 21-year tracking of hsCRP from childhood and adolescence into the fourth decade, but hsCRP was not independently associated with young adulthood carotid intimal-medial thickness (cIMT). Järvisalo et al. [22], however, showed that elevated hsCRP levels in children were associated with increased cIMT and decreased brachial artery flow-mediated dilatation, a marker of the loss of vascular flexibility. In addition, Zieske et al. [23] reported that serum hsCRP is independently associated with advanced atherosclerosis in youth.

In the current study, there was significant tracking of hsCRP across several years from age 24 to 26 (*r* = 0.66, *P* < .0001) and from age 25 to 26 (*r* = 0.71, *P* < .0001). Similarly strong long-term correlations were found from age 16 to ages 25 (*r* = 0.77, *P* < .0001) and 26 (*r* = 0.50, *P* = .02). These tracking coefficients remained significant after adjusting for multiple CVD risk factors; hsCRP at age 24 remained highly correlated with levels at age 26 (partial *r* = 0.63, *P* < .0001), as did hsCRP at age 25 with levels at age 26 (partial *r* = 0.54, *P* < .0001). Exemplifying this strong tracking of hsCRP, 24 of 43 girls (56%) with top 30% hsCRP at age 24 remained in the top 30% at age 26, while 26 of 41 girls (63%) with bottom 30% hsCRP at age 24 remained in the bottom 30% at age 26. Of 62 girls in the top 30% of the hsCRP distribution at age 25, 42 (68%) remained in the top 30% at age 26, while 49 of 72 (68%) originally in the bottom 30% at age 25 remained there at age 26. 

With exception of the report by Juonala et al. [21], tracking of hsCRP during childhood and into young adulthood has not yet been reported. With 21-year followup from childhood to young adulthood, Juonala et al. [21] reported significant tracking between childhood and adult hsCRP levels, highest in 18 years olds at study entry, *r* = 0.47 in females, 0.32 in males, and *P* < .0001 for both. For the total cohort, the 21-year tracking correlation for hsCRP was 0.29 [21]. Congruent with the findings of the current study, the association between childhood and adult hsCRP levels in Finns [21] was independent of serum lipids, blood pressure, smoking, obesity indices, and insulin. 

Significant tracking of hsCRP from childhood into young adulthood speculatively raises the issue whether childhood hsCRP can predict adult atherosclerosis or CVD events independent of traditional cardiovascular risk factors (HDL and LDL cholesterol, triglyceride, blood pressure, and obesity). In the study by Juonala et al. [21], however, childhood hsCRP was not independently associated with young adulthood carotid IMT. In 8091, subjects with hsCRP ≥ 2 mg/L randomized to placebo in the JUPITER (Rosuvastatin) trial, over a 4-year period, the intraclass correlation for repeated hsCRP measurements was 0.54 (95% CI 0.53–0.55) without adjustment and 0.50 (95% CI 0.49–0.51) after adjustment for demographic, lifestyle, and comorbidity determinants [39], Glynn et al. [39] concluded that “…concentrations of hsCRP show strong tracking, even after selection of individuals with initially high values. Without statin therapy, increased concentrations of hsCRP generally remain high over time.” 

Congruent with the findings of Soriano-Guillén et al. [20], in the current study, at age 16, hsCRP was significantly positively correlated with BMI, waist circumference, and insulin. By stepwise regression, waist circumference and BMI were the major significant (*P* < .0001) explanatory variables for hsCRP, with waist circumference accounting for 44.8% of the variance of hsCRP at age 16, and BMI accounting for 33.1% of variance of hsCRP at age 24, 34.4% at age 25, and 31.1% at age 26. The consistent, independent, and significant association of BMI with hsCRP from age 16 to 26 in our current study emphasizes the pediatric and young adult cardiovascular ramifications of obesity [1]. In a recently reported 23.9 year followup study from age 11.3 years of 4857 American Indian children without diabetes, endogenous death rates among children with the highest quartile of BMI were more than double those among children in the lowest BMI quartile (incidence-rate ratio, 2.30, 95% confidence interval 1.46 to 3.62) [1].

Our report is limited by the small number of girls (*n* = 37) that had hsCRP measurements at age 16 and then again at ages 25 and 26, thus limiting the information on tracking from adolescence into young adulthood. However, tracking data from age 24 to 26 was available in 147 girls and in 225 from age 25 to 26.

In adults, hsCRP levels have been correlated with the development of CHD in healthy men [12] and women [11, 13, 14]. In adults, hsCRP levels are associated with the traditional CHD risk factors, but also confer independent information about CHD status and future risk [10, 40]. High childhood hsCRP might be viewed as a useful index of childhood obesity that would affect progression to future premature death from endogenous causes [1] and to atherosclerotic disease [18, 41].

## Figures and Tables

**Table 1:** Simple and partial correlations of hsCRP from age 16 to age 26 years.

	At age 16	At age 24	At age 25	At age 26
hsCRP first measured	*n* = 37	*n* = 166	*n* = 366	*n* = 20

Spearman correlation with hsCRP measured at age 16	*n* = 37	*n* = 6	*n* = 22 *r* = 0.77 *P* < .0001	*n* = 21 *r* = 0.50 *P* = .021

Spearman correlation with hsCRP measured at age 24		*n* = 172	*n* = 3	*n* = 147 *r* = 0.66 *P* < .0001 partial* *n* = 139 *r* = 0.63 *P* < .0001

Spearman correlation with hsCRP measured at age 25			*n* = 391	*n* = 225 *r* = 0.71 *P* < .0001 partial* *n* = 223 *r* = 0.54 *P* < .0001

hsCRP measured at age 26				*n* = 392

*partialed by age, race, BMI, total, HDL, and LDL cholesterol, triglyceride, insulin, systolic blood pressure and diastolic blood pressure, all at the same age, and by maximum glucose measure from age 10 to age 24 years.

**Table 2:** Spearman correlations between hsCRP and cardiovascular risk disease variables at ages 16, 24, 25, and 26 years.

	Age	Race (*W* = 1, *B* = 2)	BMI	Waist	Insulin	TC	TG	HDLC	LDLC	SBP	DBP	Maximum Glucose
hsCRP at age 16	*n* = 37 *r* = −0.023 *P* = .89	*n* = 37 *r* = −0.043 *P* = .80	*n* = 37 *r* = 0.62^§^ *P* < .0001	*n* = 37 *r* = 0.63^§^ *P* < .0001	*n* = 37 *r* = 0.55^§^ *P* = .0004	*n* = 37 *r* = 0.14 *P* = .42	*n* = 37 *r* = 0.38 *P* = .022	*n* = 37 *r* = −0.13 *P* = .45	*n* = 36 *r* = 0.12 *P* = .48	*n* = 37 *r* = 0.31 *P* = .058	*n* = 37 *r* = 0.23 *P* = .17	*n* = 36 *r* = 0.35 *P* = .039

hsCRP at age 24	*n* = 171 *r* = −0.012 *P* = .88	*n* = 171 *r* = 0.072 *P* = .35	*n* = 171 *r* = 0.55^§^ *P* < .0001	*n* = 164 *r* = 0.54^§^ *P* < .0001	*n* = 171 *r* = 0.35^§^ *P* < .0001	*n* = 172 *r* = 0.19^§^ *P* = .011	*n* = 172 *r* = 0.21^§^ *P* = .0049	*n* = 172 *r* = −0.24^§^ *P* = .0015	*n* = 172 *r* = 0.21^§^ *P* = .0057	*n* = 164 *r* = 0.21^§^ *P* = .0084	*n* = 164 *r* = 0.16^§^ *P* = .040	*n* = 172 *r* = 0.20^§^ *P* = .0077

hsCRP at age 25	*n* = 391 *r* = −0.018 *P* = .73	*n* = 391 *r* = 0.086 *P* = .088	*n* = 391 *r* = 0.58^§^ *P* < .0001	*n* = 391 *r* = 0.58^§^ *P* < .0001	*n* = 391 *r* = 0.45^§^ *P* < .0001	*n* = 390 *r* = 0.26^§^ *P* < .0001	*n* = 390 *r* = 0.33^§^ *P* < .0001	*n* = 390 *r* = −0.18^§^ *P* = .0004	*n* = 390 *r* = 0.22^§^ *P* < .0001	*n* = 390 *r* = 0.22^§^ *P* < .0001	*n* = 390 *r* = 0.12^§^ *P* = .022	*n* = 385 *r* = 0.22^§^ *P* < .0001

hsCRP at age 26	*n* = 392 *r* = −0.11^§^ *P* = .028	*n* = 392 *r* = 0.16 *P* = .75	*n* = 392 *r* = 0.56^§^ *P* < .0001	*n* = 392 *r* = 0.55^§^ *P* < .0001	*n* = 391 *r* = 0.35^§^ *P* < .0001	*n* = 391 *r* = 0.18^§^ *P* = .0003	*n* = 391 *r* = 0.37^§^ *P* < .0001	*n* = 391 *r* = −0.17^§^ *P* = .0005	*n* = 391 *r* = 0.14^§^ *P* = .0049	*n* = 392 *r* = 0.22^§^ *P* < .0001	*n* = 392 *r* = 0.13^§^ *P* = .0090	*n* = 388 *r* = 0.24^§^ *P* < .0001

^§^significant by Benjamini false discover rate controlling (*P* < .05, for 12 correlations for each hsCRP measure)

Waist: waist circumference, TC: total cholesterol, TG: triglyceride, HDLC: high-density lipoprotein cholesterol, LDLC: Low-density lipoprotein cholesterol, SBP: systolic blood pressure, DBP: diastolic blood pressure. Maximum glucose: maximum of all available glucose measures from age 10 to age 24.

**Table 3:** Stepwise regression of hsCRP at ages 16, 24, 25, and 26 hsCRP are log transformed. Explanatory variables include race, age, BMI, waist circumference, insulin, TC, TG, HDLC, LDLC, systolic, and diastolic blood pressures all at the age when hsCRP measured, and maximum glucose (from age 10 to age 24). Significant levels: SLE = 0.15, SLS =  .05 in stepwise selection.

Dependent variable	Significant explanatory variable	sign	Partial *R* ^2^	*P*
hsCRP at age 16 (35 observations used)	waist circumference	+	44.8%	<.0001

hsCRP at age 24 (164 observations used)	BMI	+	33.1%	<.0001
	TC	+	2.5%	.014

hsCRP at age 25 (383 observations used)	BMI	+	34.4%	<.0001
	TC	+	1.9%	.0008
	waist circumference	+	0.8%	.030

hsCRP at age 26 (386 observations used)	BMI	+	31.1%	<.0001
	TG	+	3.5%	.0002
	Age	−	2.1%	.0010
	Race (*W* = 1, *B* = 2)	−	1.1%	.0092

TC: total cholesterol, TG: triglyceride, HDLC: HDL cholesterol, LDLC: LDL cholesterol.

**Table 4:** Spearman correlations between changes in hsCRP (log transformed) and changes in cardiovascular disease risk variables from ages 24 to 26 and from ages 25 to 26.

	Change in Age	Race (*W* = 1, *B* = 2)	Change in BMI	Change in Waist	Change in TC	Change in TG	Change in HDLC	Change in LDLC	Change in SBP	Change in DBP
Change in hsCRP from age 24 to 26	*n* = 147 *r* = −0.051 *P* = .54	*n* = 147 *r* = −0.053 *P* = .53	*n* = 147 *r* = 0.27^#^ *P* = .0008	*n* = 140 *r* = 0.20* *P* = .020	*n* = 147 *r* = 0.27^#^ *P* = .0010	*n* = 147 *r* = 0.21* *P* = .014	*n* = 147 *r* = 0.43 *P* = .61	*n* = 147 *r* = 0.23^#^ *P* = .0049	*n* = 140 *r* = 0.090 *P* = .29	*n* = 140 *r* = 0.12 *P* = .17

Change in hsCRP from age 25 to 26	*n* = 225 *r* = 0.26 *P* = .70	*n* = 225 *r* = −0.089 *P* = .18	*n* = 225 *r* = 0.36^§^ *P* < .0001	*n* = 225 *r* = 0.33^§^ *P* < .0001	*n* = 224 *r* = 0.16* *P* = .017	*n* = 224 *r* = 0.28^§^ *P* < .0001	*n* = 224 *r* = −0.069 *P* = .30	*n* = 224 *r* = 0.079 *P* = .24	*n* = 224 *r* = 0.063 *P* = .34	*n* = 224 *r* = −0.10 *P* = .14

*P < .05, ^#^ *P* < .01, ^†^ *P* < .001, ^§^ *P* ≤ 0001.

Waist: waist circumference, TC: total cholesterol, TG: triglyceride, HDLC: HDL cholesterol, LDLC: LDL cholesterol, SBP: systolic blood pressure, DBP: diastolic blood pressure.

**Table 5:** Change in hsCRP (logarithm transformed) from age 24 to age 26 and from age 25 to 26.

Dependent variable	Significant explanatory variable	sign	Partial *R* ^2^	*P*
Change in hsCRP from age 24 to age 26 (139 observations used)	Change in TC	+	12.3%	<.0001
	Change in BMI	+	6.6%	.0012

Change in hsCRP from age 25 to age 26 (223 observations used)	Change in BMI	+	8.5%	.0001
	Change in TG	+	3.3%	.0045

Dependent variable: hsCRP (logarithm transformed) change.

Explanatory variables include: race, change in age, change in BMI, changes in waist circumference, changes in TC, TG, HDLC, and LDLC, and change in blood pressure during the same period.

Significant levels: SLE  =  .15, SLS  =  .05.
